# Associations of perceived family economy, registry-based parental education and income with adolescent psychological distress: the Young-HUNT cross-sectional studies 2006–2008 and 2017–2019

**DOI:** 10.1136/bmjopen-2025-111941

**Published:** 2026-06-10

**Authors:** Karoline Louise Imingen Selvik, Bent Martin Eliassen Strandås, Craig A Olsson, Tonje Braaten, Ottar Bjerkeset

**Affiliations:** 1 Faculty of Nursing and Health Sciences, Nord University, Bodø, Norway; 2 Department of Mental Health and Addiction Medicine, Nordlands Hospital Trust, Bodø, Norway; 3 Deakin Lifespan Institute, School of Psychology, Faculty of Health, Deakin University, Burwood, Victoria, Australia; 4 LifeCourse, Murdoch Children's Research Institute, Royal Children's Hospital, Melbourne, Victoria, Australia; 5 Department of Community Medicine, UiT The Arctic University of Norway, Tromsø, Norway; 6 Faculty of Nursing and Health Sciences, Nord University, Levanger, Norway

**Keywords:** MENTAL HEALTH, Adolescent, Child & adolescent psychiatry

## Abstract

**Abstract:**

**Objectives:**

This study aimed to investigate the associations of adolescents’ self-reported family financial stress, registry-based parental household income and parental education with adolescent anxiety and depression symptoms. Additionally, we adjust these associations for parental anxiety and depression symptoms and examine potential secular changes in these associations.

**Design:**

Family linkage study, using two cross-sectional population-based health studies, the Young-HUNT study and the HUNT study. Registry-based data from Statistics Norway (SSB).

**Setting:**

Northern part of Trøndelag County, Norway.

**Participants:**

Adolescent (aged 13–19 years) participating in The Young-HUNT3 Survey (2006–2008, n=8199) and The Young-HUNT4 Survey (2017–2019, n=8066) and their parents participating in The HUNT3 Survey (2006–2008, n=50 800) and the HUNT4 Survey (2017–2019, n=56 042).

**Measurements:**

Adolescent anxiety and depression symptoms were assessed by a short version of the Hopkins Symptom Checklist (HSCL), the five-item HSCL-5. Self-reported family financial stress was measured using a single-item question. Parental anxiety and depression were assessed by the 14-item Hospital Anxiety and Depression Rating Scale (total HADS score). Parental income and parental education were obtained from SSB. We use a multilevel mixed-effects generalised linear model.

**Results:**

Adolescents who perceived their family financial stress as worse than others reported a higher SCL-5 total score compared with those with self-perceived average financial stress. The relative differences ranged from 1.16 (95% CI 1.09 to 1.23) in boys to 1.24 (95% CI 1.17 to 1.31) in girls. In contrast, little or no association was found between parental registry-based income or educational level and adolescents’ mean SCL-5 total scores. Adjusting for parental HADS scores did not alter the estimates. With a few exceptions for girls, there was no evidence for a secular change in these associations.

**Conclusion:**

Self-perceived family financial stress, but not registry-based parental income and education, was associated with elevated anxiety and depression symptom levels in adolescents, and findings were essentially the same in Young-HUNT3 and Young-HUNT4. These findings underscore the importance of incorporating multiple measures of socioeconomic status when investigating socioeconomic inequalities in adolescent mental health.

**STRENGTHS AND LIMITATIONS OF THIS STUDY**

- The HUNT and the Young-HUNT studies are renowned population studies with large sample sizes and acceptable (HUNT) to high (Young-HUNT) participation rates.
- The family linkage within HUNT and external linkage to national registry data provide valid information about mental health in both parents and offspring, and family socioeconomic status (SES).
- Psychological distress was measured using well-validated scales in both adolescents (Hopkins Symptom Checklist) and their parents (Hospital Anxiety and Depression Rating Scale).
- Geographically, these studies were conducted in a predominantly rural area with a few smaller towns, and our results may not be generalisable to larger cities with greater SES inequalities in Norway.

## Introduction

 Globally, a striking increase in adolescent self-reported psychological distress has repeatedly been reported over the last three decades, particularly internalising symptoms among girls.^1^ Similar trends have been reported in the Nordic countries, including Norway.^2^,^5^ A wide range of potential explanations for this secular trend have been proposed, including increased mental health awareness, reduced stigma, academic pressure, excessive smartphone use, unregulated social media, pandemics and climate change.^1^ Simultaneously, the prevalence and impact of mental disorders in 10–19 years olds increased markedly from 1990 to 2021.^1 6 7^ Approximately half of all mental disorders emerge during childhood or adolescence,^8^ with many persisting into adulthood.^9^ Depression, anxiety and behavioural disorders are among the leading causes of illness and disability among adolescents, accounting for 36% of the Years Lived with Disability among individuals aged 20–29.^10^ Collectively, these findings highlight the formative years of adolescence as a critical period for prevention, early detection and improved treatment.

Mental health problems and disorders in young people arise from a complex interplay between biological, psychological and social risk factors.^11^ Low family socioeconomic status (SES), often defined by low household income and/or low parental education, has been linked to poor childhood mental health, especially externalising disorders.^12^ Other studies have also identified strong associations between self-reported SES/concerns about family finances and adolescent mental health.^13 14^ An international trend study from 2002 to 2010 from 34 high-income countries reported an increase in socioeconomic inequalities in adolescent mental health, particularly in countries with high national income inequalities.^15^ A study using data from three waves of the Norwegian HUNT Study from 1995 to 2019 reported a small increase in socioeconomic inequalities in adolescent mental health,^16^ while another Norwegian study using five waves of the national cross-sectional Ungdata survey found no such increase.^17^

The family stress model describes how perceived economic difficulties can create parental stress and behavioural changes that negatively impact family life, increasing the risk of worry and anxiety in children and adolescents.^18^ In contrast, the family investment model centres on how a lack of objective family material resources can limit children’s opportunities to maintain health and achieve cognitive and educational development.^19^ Additionally, stress related to social status may contribute to socioeconomic differences in self-rated health.^20^ Furthermore, the intergenerational transmission of mental health problems appears to be strongest in families with low SES.^21^ Parental mental disorders are also a major confounder in the relationship between parental SES and children’s mental health problems.^12^

In this study, we aim to investigate the associations of adolescents’ self-reported family financial stress and registry-based parental income and education with adolescent anxiety and depression symptom level. Secondly, we examined potential secular changes in these associations. Data were drawn from the Young-HUNT study and the HUNT Study in 2006–2008 and 2017–2019, and Statistics Norway (SSB).

## Methods

### Data and study population

We used data from two repeated cross-sectional population-based health studies, the Young-HUNT study (adolescents) and the HUNT study (parents/adults), along with registry data from SSB. The health studies were conducted in the northern part of Trøndelag County (former Nord-Trøndelag), Norway.^22^,^24^

Adolescents recruited to this study consisted of two unique samples who participated in the Young-HUNT3 Survey (2006–2008, n=8199, 78.4% response rate) and the Young-HUNT4 Survey (2017–2019, n=8066, 76.0% response rate). These studies are school-based surveys, and invitations were based on schools’ class lists of all registered pupils primarily aged 13–19 years in all lower and upper secondary schools in the county. In the Young-HUNT3 survey (hereinafter referred to as Young-HUNT3), 66 schools participated, and in the Young-HUNT4 survey (hereinafter referred to as Young-HUNT4), 67 schools participated. Participants aged 16 and older provided informed written consent, while parents provided written consent for participants younger than 16 years. Adolescents completed the self-report questionnaire on paper in Young-HUNT3 and online in Young-HUNT4 during school hours. Pupils who were absent on the day of the survey received the survey by post. Furthermore, clinical examinations were conducted on more than half of the participants.^23 24^

All inhabitants in Nord-Trøndelag aged 20 years and older were invited to participate in each survey of the HUNT Study. Participants completed a self-report questionnaire on paper or web-based. Subsamples were invited to a short interview, new questionnaires, clinical examinations and biological sampling.^22^ Unique project-specific ID numbers from HUNT were used to link data from Young-HUNT with parental data from the HUNT3 Survey (2006–2008, n=50 800, 54.1% response rate), the HUNT4 Survey (2017–2019, n=56 042, 54.0% response rate) and registry data from SSB, including a unique family number identifying participants with mutual parents.

**Figure 1:**
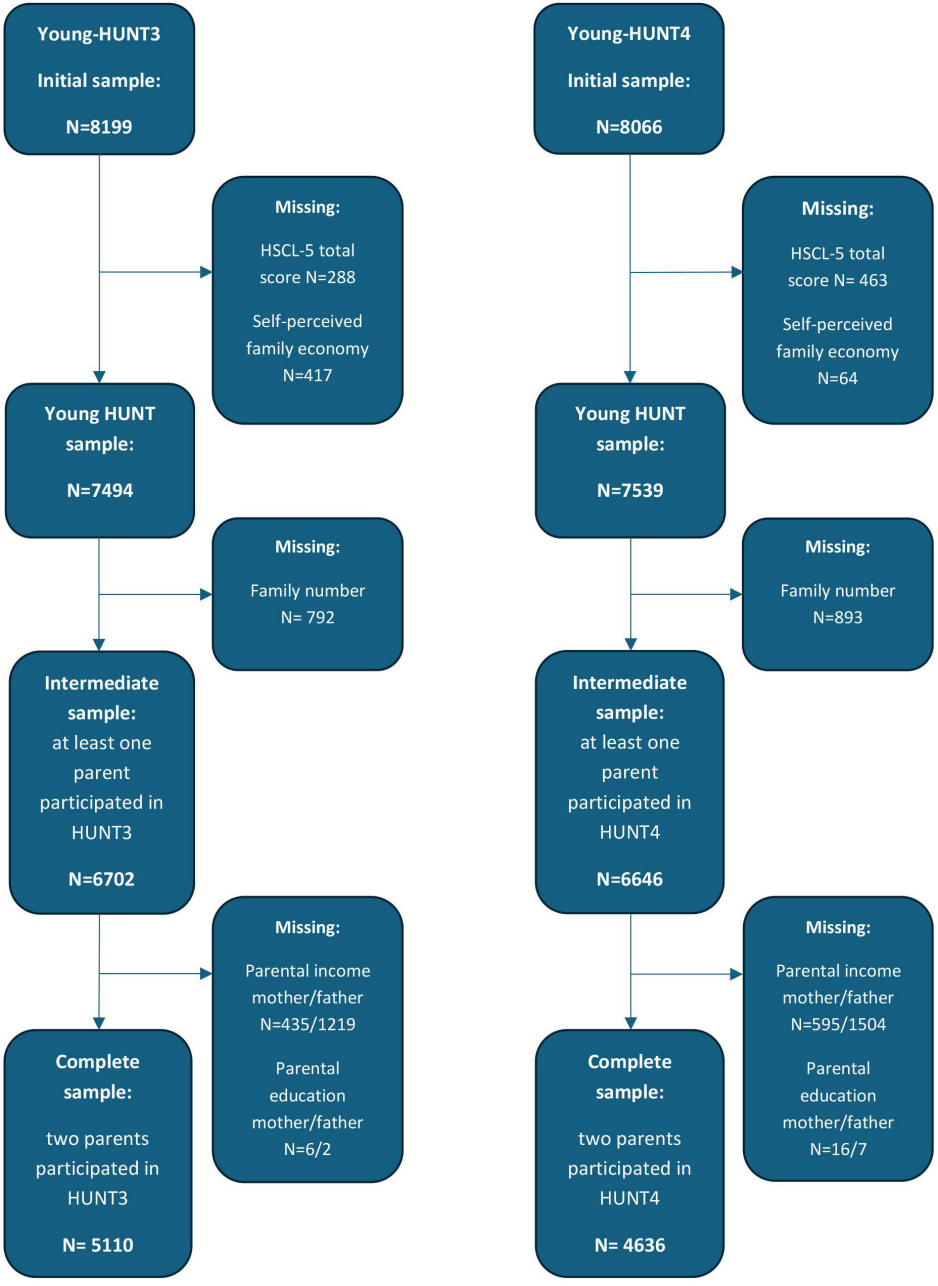
Flowcharts: participation and exclusion in the Young-HUNT 3 Survey (2006–2008) and the Young-HUNT4 Survey (2017–2019). HSCL, Hopkins Symptom Checklist.

From the initial sample of 16 265 adolescents, we excluded those with missing information on the SCL-5 total score (n=751) and self-perceived family financial stress (n=481), constituting the “Young-HUNT sample”. We further excluded adolescents with parental non-participation in the concurrent HUNT3- or HUNT4 surveys, resulting in an “Intermediate sample” of 13 348 adolescents. Finally, we retained adolescents with both parents participating in HUNT3 or in HUNT4. This resulted in the “Complete sample” of 9746 participants: 5110 adolescents (50.4% girls and 49.6% boys) from Young-HUNT3 and 4636 adolescents (50.2% girls and 49.8% boys) from Young-HUNT4 (figure 1, table 1). We adjust for parental HADS-T in a subsample of the “Complete sample”; “the HADS sample”; 41% in Young-HUNT3 (n=2077) and 37% in Young-HUNT4 (n=1704).

**Table 1:** Descriptive characteristics: the Young-HUNT3 Survey (2006–2008) and the Young-HUNT4 Survey (2017–2019): Young- HUNT-*, intermediate-† and complete sample‡

Survey	Young-HUNT3			Young-HUNT4		
Sample	Young-HUNT*	Intermediate†	Complete‡	Young HUNT*	Intermediate†	Complete‡
Sample size (N)	7494	6702	5110	7539	6646	4636
Girls (N (%))	3856 (51.5)	3433 (51.2)	2576 (50.4)	3882 (51.5)	3439 (51.8)	2328 (50.2)
Mean age (SD)	15.9 (1.8)	15.9 (1.8)	16.00 (1.8)	16.1 (1.8)	16.1 (1.8)	16.1 (1.8)
Mean HSCL-5 total score (SD)	1.7 (0.6)	1.6 (0.6)	1.6 (0.6)	1.9 (0.8)	1.9 (0.8)	1.9 (0.7)
Worse self-perceived family financial stress (N (%))	396 (10.3)	337 (9.8)	205 (8.0)	358 (9.2)	301 (8.8)	162 (7.0)
Boys (N (%))	3638 (48.6)	3269 (48.8)	2534 (49.6)	3657 (48.5)	3207 (48.3)	2308 (49.8)
Mean age (SD)	15.9 (1.7)	15.9 (1.7)	16.0 (1.7)	16.1 (1.8)	16.1 (1.8)	16.1 (1.8)
Mean HSCL-5 total score (SD)	1.3 (0.4)	1.3 (0.4)	1.3 (0.4)	1.4 (0.5)	1.4 (0.5)	1.4 (0.5)
Worse self-perceived family financial stress (N (%))	304 (8.4)	254 (7.8)	170 (6.7)	241 (6.6)	196 (6.1)	106 (4.6)

* Adolescents participating in the Young-HUNT3 Survey and the Young-HUNT4 Survey.

† Adolescents with one parent participated in the HUNT3 or HUNT4 Survey.

‡ Adolescents with two parents participated in the HUNT3 or HUNT4 Survey.

HSCL, Hopkins Symptom Checklist.

### Measurements

#### Dependent variable

Self-reported psychological distress (anxiety and depression symptoms) was assessed by a validated, reliable and widely used Norwegian short version of the Hopkins Symptom Checklist (HSCL), the five-item HSCL-5 in Young-HUNT3 and Young-HUNT4.^25^,^27^ HSCL-5 measures the frequency of five psychological symptoms over the past 2 weeks: feeling tense, anxious, hopeless, sad and worried. Each question used a four-point Likert scale ranging from ‘not bothered’ (1 point) to ‘very much bothered’ (4 points). The mean HSCL-5 score was calculated by dividing the total sum by five, with scores ranging from 1 to 4. Missing data on HSCL-5 in both surveys ranged from 3.5% to 5.7%.

#### Independent variables

##### Self-perceived family financial stress

Self-perceived family financial stress was measured in both Young-HUNT Surveys using the question *‘How well off do you think your family is compared with most others?*’. The question originates from the WHO collaborative cross-national Health Behaviour in School-aged Children study in Europe.^28^ Response options included: *“About the same as most others”, “Better financial situation” and “Worse financial situation”*. All three response categories were retained. Missing was 5.2% (girls) and 8.5% (boys) in Young-HUNT3, and 1.2% (girls) and 1.8% (boys) in Young-HUNT4.

##### Parental income and educational level

Parental income was assessed by total household income, after tax, divided by the number of family members in the household. This data was obtained from SSB for the same year the youth participated in Young-HUNT. We divided total household income into low, middle and high income using tertiles separate for Young-HUNT3 (2006–2008) and Young-HUNT4 (2017–2019) (online supplemental table 1). Missing in both surveys due to non-attendance in HUNT ranged from 14.9% (mothers’ income) to 31.9% (fathers’ income).

Parental educational level was obtained from SSB for the same year the adolescent participated in Young-HUNT3 and Young-HUNT4. Mothers’ and fathers’ education level was divided into primary (<10 years), secondary (10–14 years) and tertiary (>14 years) education (online supplemental table 2). Missing data due to parental non-attendance in the HUNT3 and HUNT4 Surveys ranged from 14.7% (mothers’ education) to 31.5% (fathers’ education).

### Additional variables

#### Parental anxiety and depression symptoms

Parental anxiety and depression symptoms were assessed using the validated, reliable and widely used 14-item Hospital Anxiety and Depression rating Scale (HADS) in both HUNT Surveys.^29 30^ HADS measures symptoms over the past week on a 7-item anxiety (HADS-A) and 7-item depression subscale (HADS-D). Each question used a four-point Likert scale from ‘not at all’ (0 points) to ‘nearly all the time’ (3 points).^29^ We used the total HADS extrapolated score (HADS-T), the sum of all HADS items (range 0 to 42). If 11 or more items were answered, the score was extrapolated by multiplying the sum by 14/11, 14/12 or 14/13, respectively. HADS-T was set to missing if 10 or fewer items were answered. HADS-T was used as a continuous score.

#### Parental country of origin

Parental country of origin was obtained from SSB and categorised as Norway, EU/EEA/European countries outside EU and outside Europe. Missing data for mothers’ and fathers’ country of origin ranged from 14,5% for mothers to 31.3% for fathers.

### Statistical analyses

We computed gender- and survey-specific characteristics of all samples. Continuous variables are presented as means with SD, and categorical variables as frequencies with proportions. Associations between self-perceived family financial stress and parental income and education, and adolescent anxiety and depression symptom levels were examined using a multilevel mixed-effects generalised linear model with random intercept to account for correlation between siblings. A gamma distribution with a log link function was used to estimate the ratio of predicted means as a measure of relative differences (RD) with 95% CIs, stratified by gender. Analyses were adjusted for continuous age. Since the number of missing HADS-T scores was relatively high, despite extrapolation, we adjusted for parental HADS-T only in a subsample of the “Complete sample”; the “HADS-sample”.

Parental country of origin was used in a sensitivity analysis to examine potential changes in the association between the three SES factors and symptom level (mean HSCL-5 total score) after excluding participants with parents of non-Norwegian background from the “Complete sample”.

Secular change from Young-HUNT3 to Young-HUNT4 was examined using the likelihood ratio test to compare models with and without terms for interaction between survey and SES factors.

All statistical analyses were performed using Stata, V.17.0.

## Results

### Descriptive results

Table 1 presents the characteristics of the “Young-HUNT”, “Intermediate” and “Complete sample”. The mean age was approximately 16.0 years for boys and girls in both surveys. The mean HSCL-5 total score, with the maximum possible score of 4, was 1.6 for girls in Young-HUNT3 and 1.9 points in Young-HUNT4. The respective numbers for boys were 1.3 and 1.4 points. Furthermore, we saw a decrease in the proportion of adolescents reporting self-perceived family financial stress, from 8% and 6.7% in Young HUNT3 to 7% and 4.6% in Young-HUNT4, in girls and boys in the “Complete sample”, respectively (table 1). The characteristics of the “HADS sample” mirrored those of the other samples, with the notable exception that a smaller proportion of adolescents reported worse self-perceived family financial stress in the “HADS sample” (online supplemental table 3).

In Young-HUNT3 and YoungHUNT4, the median household income per family member (in NOK) varied between 245 585 (mothers’ income in boys) and 260 199 (fathers’ income in girls), and 385 565 (mothers’ income in girls) and 400 172 (fathers’ income in girls), respectively (table 2).

**Table 2:** Descriptive characteristics: the Young-HUNT3 Survey (2006–2008) and the Young-HUNT4 Survey (2017–2019): intermediate-* and complete sample†

Survey	Young-HUNT3:		Young-HUNT4:	
Sample	Intermediate*	Complete†	Intermediate*	Complete†
Sample (N)	6702	5110	6646	4636
Girls	3433 (51.2)	2576 (50.4)	3439 (51.8)	2328 (50.2)
Median ‡income mother	248 549	252 587	374 737	385 565
Median ‡income father	259 650	260 199	395 476	400 172
Education mother: tertiary education (N (%))	1207 (37.6)	995 (38.6)	1725 (55.2)	1338 (57.5)
Education father: tertiary education (N (%))	710 (25.4)	659 (25.6)	872 (33.2)	774 (33.3)
Background mother: Norway (N (%))	3008 (93.5)	2439 (94.7)	2867 (91.4)	2178 (93.6)
Background father: Norway (N (%))	2658 (95.2)	2455 (95.3)	2487 (94.5)	2208 (94.9)
Boys	3269 (48.8)	2534 (49.6)	3207 (48.3)	2308 (49.8)
Median ‡income mother	244 506	245 585	378 993	386 021
Median ‡income father	251 030	251 369	393 094	395 268
Education mother: tertiary education (N (%))	1209 (39.3)	1007 (39.7)	1682 (57.3)	1366 (59.2)
Education father: tertiary education (N (%))	704 (25.8)	658 (26.0)	853 (33.4)	788 (34.1)
Background mother: Norway (N (%))	2894 (94.0)	2393 (94.4)	2693 (91.3)	2144 (92.9)
Background father: Norway (N (%))	2624 (96.0)	2437 (96.2)	2380 (93.1)	2156 (93.4)

* Adolescents with one parent participated in the HUNT3 or HUNT4 Survey.

† Adolescents with two parents participated in the HUNT3 or HUNT4 Survey.

‡ Income=total household income, after tax, divided by the number of family members in the household.

The proportion of girls in the “Complete sample” with highly educated mothers increased from 38.6% (Young-HUNT3) to 57.5% (Young-HUNT4). Respective numbers for boys were 39.7%–59.2%. The corresponding numbers for girls with highly educated fathers were 25.6% (Young-HUNT3) and 33.3% (Young-HUNT4). The increase for boys was 26.0%–34.1% (table 2).

### Main results

#### Self-perceived family financial stress

In both surveys, girls and boys who perceived their family financial stress as worse than others reported higher mean HSCL-5 total scores compared with those who rated their family’s financial stress as similar or the same as others (table 3). In the “Complete sample”, the RD ranged from 1.16 in boys (95% CI 1.09 to 1.23) to 1.24 in girls (95% CI 1.17 to 1.31) (table 3). Adjusting for parental mental health did not alter the results (online supplemental table 4). There was no indication of a secular change in the estimates between the two surveys.

**Table 3:** Relative difference: SES and psychological distress among girls and boys (13–19 years) in the Young-HUNT3 Survey (2006–2008) and the Young-HUNT4 Survey (2017–2019), Young-HUNT-*, intermediate† and complete sample‡

Survey	The Young-HUNT3 survey			The Young-HUNT4 survey		
Sample	Young-HUNT*	Intermediate†	Complete‡	Young-HUNT*	Intermediate†	Complete‡
	RD (95% CI)	RD (95% CI)	RD (95% CI)	RD (95% CI)	RD (95% CI)	RD (95% CI)
Girls						
Self-perceived family financial stress						
Worse (ref same)	1.23 (1.19 to 1.28)	1.23 (1.19 to 1.28)	1.21 (1.15 to 1.27)	1.27 (1.21 to 1.32)	1.24 (1.17 to 1.31)	1.24 (1.17 to 1.31)
Better (ref same)	1.03 (0.99 to 1.06)	1.03 (0.99 to 1.06)	1.03 (0.99 to 1.06)	1.01 (0.98 to 1.05)	1.03 (0.99 to 1.07)	1.03 (0.99 to 1.07)
Income mother§						
Middle (ref low)		1.01 (0.98 to 1.04)	1.04 (1.00 to 1.09)		0.98 (0.95 to 1.01)	0.97 (0.92 to 1.01)
High (ref low)		0.99 (0.96 to 1.02)	0.95 (0.91 to 1.00)		0.96 (0.93 to 0.99)	0.94 (0.89 to 0.99)
Income father§						
Middle (ref low)		1.02 (0.99 to 1.05)	1.01 (0.97 to 1.05)		0.98 (0.94 to 1.01)	1.00 (0.95 to 1.04)
High (ref low)		1.05 (1.02 to 1.09)	1.10 (1.05 to 1.15)		0.95 (0.92 to 0.99)	1.00 (0.95 to 1.05)
Education mother						
Secondary education (ref primary)		0.99 (0.96 to 1.03)	1.00 (0.97 to 1.05)		0.96 (0.91 to 1.01)	0.97 (0.90 to 1.03)
Tertiary education (ref primary)		1.00 (0.97 to 1.04)	1.02 (0.97 to 1.06)		0.93 (0.88 to 0.98)	0.93 (0.87 to 0.99)
Education father						
Secondary education (ref primary)		0.98 (0.95 to 1.02)	0.99 (0.95 to 1.03)		0.98 (0.93 to 1.03)	1.00 (0.94 to 1.05)
Tertiary education (ref primary)		0.99 (0.95 to 1.03)	0.98 (0.94 to 1.03)		0.95 (0.90 to 1.00)	0.97 (0.91 to 1.03)
Boys						
Self-perceived family financial stress						
Worse (ref same)	1.25 (1.20 to 1.29)	1.25 (1.20 to 1.29)	1.23 (1.18 to 1.28)	1.19 (1.14 to 1.25)	1.19 (1.14 to 1.25)	1.16 (1.09 to 1.23)
Better (ref same)	1.04 (1.02 to 1.06)	1.04 (1.02 to 1.06)	1.03 (1.00 to 1.05)	1.02 (0.99 to 1.05)	1.02 (0.99 to 1.04)	1.02 (0.99 to 1.05)
Income mother§						
Middle (ref low)		0.99 (0.96 to 1.01)	1.01 (0.97 to 1.04)		1.01 (0.98 to 1.04)	0.99 (0.95 to 1.03)
High (ref low)		0.99 (0.97 to 1.02)	0.98 (0.94 to 1.02)		0.99 (0.96 to 1.02)	0.99 (0.94 to 1.04)
Income father§						
Middle (ref low)		0.98 (0.96 to 1.01)	0.98 (0.95 to 1.02)		0.97 (0.94 to 1.01)	0.99 (0.95 to 1.03)
High (ref low)		1.01 (0.98 to 1.04)	1.03 (0.99 to 1.07)		0.99 (0.95 to 1.02)	1.01 (0.96 to 1.06)
Education mother						
Secondary education (ref primary)		0.98 (0.95 to 1.01)	0.98 (0.95 to 1.02)		0.99 (0.95 to 1.04)	0.98 (0.92 to 1.04)
Tertiary education (ref primary)		0.99 (0.95 to 1.01)	0.99 (0.96 to 1.03)		1.00 (0.95 to 1.05)	0.98 (0.93 to 1.04)
Education father						
Secondary education (ref primary)		1.01 (0.97 to 1.04)	1.01 (0.98 to 1.04)		0.99 (0.94 to 1.03)	0.99 (0.94 to 1.05)
Tertiary education (ref primary)		1.00 (0.96 to 1.03)	1.00 (0.96 to 1.04)		0.99 (0.94 to 1.04)	1.00 (0.94 to 1.06)

* Adolescents participating in the Young-HUNT3 Survey and the Young-HUNT4 Survey.

† Adolescents with one parent participated in the HUNT3 or HUNT4 Survey; mother and father in separate models.

‡ Adolescents with two parents participated in the HUNT3 or HUNT4 Survey; mother and father in the same model.

§ Income=total household income, after tax, divided by the number of family members in the household.

RD, Relative difference.

#### Parental income

Overall, the analysis provided little statistical evidence for an association between registry-based parental income and adolescent psychological distress in either Young-HUNT3 or Young-HUNT4. Statistically significant associations were only observed in girls with high-income fathers (RD=1.10, 95% CI 1.05 to 1.15) in Young HUNT3, and in girls with high-income mothers (RD=0.94, 95% CI 0.89 to 0.99) in Young-HUNT4 (table 3). Adjustment for parental mental health did not alter the estimates (online supplemental table 4). Overall, the estimates were stable across surveys in both boys and girls, yet we observed a statistically significant secular change in the associations with both parents’ income among girls (p<0.001).

#### Parental education

Similarly, adolescents of parents with the lowest level of education exhibited the same mean HSCL-5 total score as those with secondary or tertiary levels of education in both surveys. The only statistically significant association was observed in girls of mothers with tertiary education in Young-HUNT4 (RD=0.93, 95% CI 0.87 to 0.99) (table 3). Estimates largely remained the same after adjustment for parental mental health (online supplemental table 4). Estimates were stable across surveys, though the change in the association with mother’s education was significant among girls (p=0.002).

#### Sensitivity analysis

Repeating the main analysis in the “Complete sample” after excluding participants with parents of non-Norwegian backgrounds did not alter the estimates (online supplemental table 5).

## Discussion

### Key results

In two population-based surveys of Norwegian adolescents and their parents (2006–2008 and 2017–2019), we found that adolescents who perceived worse family financial stress had, on average, a 16%–24% increased predicted mean HSCL-5 total score compared with those with self-perceived average financial stress. In contrast, there was little or no support for an association between parental registry-based income and educational level with the mean HSCL-5 total score in their offspring. Further analyses adjusting for parental mental health did not alter the estimates. With a few exceptions for girls, there was no evidence for a secular change in these associations.

### Interpretation/main discussion

Our main findings are consistent with results from a study of adolescents in Sweden using repeated cross-sectional data collected in four waves during 2002–2014. They found that socioeconomic inequalities in adolescent self-reported health defined by subjective SES (one single-item question: ‘How would you describe the economic situation in your family?’) were larger than when assessed by the Family Affluence Scale (FAS) (a proxy for objective SES).^14^ Similarly, a Nordic study from 2002 to 2018 showed that the association between perceived family wealth and mental health (measured by life satisfaction and subjective health complaints) was stronger than the association between FAS and mental health.^31^

In the present study, we found no consistent secular change in the association of self-perceived family financial stress and registry-based parental SES markers with adolescent anxiety and depression symptoms from 2006 to 2008 to 2017–2019. These findings are consistent with a previous national Norwegian study.^17^ However, using Young-HUNT survey data, Aasan *et al* observed a minor increase in socioeconomic inequalities in adolescent mental health during the same period, which may be attributed to larger sample sizes, as their analyses were not restricted to adolescents whose parents participated in the HUNT surveys.^16^

A meta-analysis investigated the association and the magnitude of the association between subjective SES and different health outcomes (self-rated health, mental health, physical health and health behaviours) during adolescence (12–19 years). They found that subjective SES measures were associated with mental health outcomes, self-rated health and general health symptoms. The relationship between subjective SES and mental health demonstrated the largest effect size.^32^ Findings support the use of subjective SES in the examination of social disparities in mental health.

A study in 27 welfare states showed that adolescents in countries with low income inequality and a social democratic regime, such as Norway, were associated with lower levels of psychosomatic health complaints and a weaker association between FAS (a proxy for objective SES) and psychosomatic health complaints compared with adolescents from countries with higher income inequality and with a liberal welfare tradition.^33^ Low levels of income inequality in Norway may therefore partly explain the lack of statistical evidence between objective parental SES measures and the mean adolescent HSCL-5 score in our study. Furthermore, a Norwegian study from 2012 by Bøe *et al*, investigating the association between subjective (perceived economic well-being) and objective SES measures (household income) and mental health in adolescents (aged 16–19), found that the mental health benefits associated with higher income appeared to depend on the adolescents’ perceptions of their family’s relative economic position. In their study, the influence of household income outweighed perceptions of economic well-being only at very low household income levels. Of note, subjective SES was the core factor associated with peer and emotional problems for most participants.^34^ This may reflect that social ranking and social comparison are important mechanisms in explaining SES inequality in mental health in a high-income country. Subjective SES may also reflect factors such as family wealth, neighbourhood socioeconomic conditions, past economic experiences, family composition and the employment status of parents.^34^

Notably, a systematic review by Reiss^12^ found that parental mental disorders confounded the relationship between parental SES and children’s mental health problems. In our study, we adjusted the associations between self-perceived family financial stress and mean HSCL-5 total scores for parental anxiety and depression symptoms in a small subsample (the “HADS sample”). In the “HADS sample”, fewer adolescents reported worse self-perceived family financial stress compared with the original samples, yet the results remained unchanged. This may be due to the use of parental self-reported symptom levels rather than parental mental health disorders, the latter indicating more severe conditions.

Overall, our findings support existing evidence that subjective and objective SES measures represent different sets of health-relevant resources, and their effect on adolescent mental health may be different. This underscores the importance of using multiple measures of SES when investigating socioeconomic inequalities in adolescent mental health, particularly in high-income countries like Norway.

### Strengths and limitations

The Young-HUNT/HUNT Studies are renowned population studies with large sample sizes and an acceptable (HUNT) to high (Young-HUNT) participation rates. Psychological distress was measured using well-validated scales in both adolescents and their parents (HSCL-5 and HADS, respectively). Additionally, the family linkage within HUNT and external linkage to national registry data gives valid information about mental health in both parents and offspring, and family SES.

Geographically, these studies were conducted in a predominantly rural area with a few smaller towns, but no large cities. The population is relatively ethnically homogeneous and characterised by slightly lower educational and income levels than Norway as a whole, yet generally it is considered nationally representative.^22 23^ Nevertheless, our results may not be generalisable to more urban areas with greater ethnic, social and economic inequalities in Norway.

The cross-sectional design of this study precludes causal inferences. The relationship between subjective SES and mental health may be bidirectional. Additionally, measures of subjective SES may capture adolescents who are simultaneously exposed to additional risk factors alongside low social status, thereby increasing their susceptibility to stress and negative health outcomes.^32^

This study did not include externalising symptoms in adolescents, which some studies have found to be more strongly associated with low SES compared with internalising symptoms.^12^ This might have led to an underestimation of socioeconomic differences in mental health in our study.

Non-responder studies in most population surveys, including HUNT/Young-HUNT, indicate that non-participants generally have less healthy lifestyles, more chronic conditions and psychosocial problems.^22 24^ Additionally, excluding adolescents whose parents did not participate in the HUNT Study may have led to an underestimation of socioeconomic differences in mental health.

## Conclusion

Self-perceived family financial stress was associated with 16%–24% increased mean HSCL-5 scores in 13–19 year-old girls and boys who participated in Young HUNT3 (2006–2008) and Young HUNT4 (2017–2019). In contrast, there was little or no support for an association between objective SES markers (parental income and education) and mean HSCL-5 score. Adjusting for parental anxiety and depression symptoms did not alter the estimates. Additionally, aside from a few exceptions in some sub-analyses for girls, there was little evidence of secular changes in these associations from Young-HUNT3 to Young-HUNT4. Taken together, these findings highlight the importance of incorporating multiple measures of SES when investigating socioeconomic inequalities in adolescent mental health in high-income countries. Subjective SES measures may capture unique aspects of adolescents’ lived experiences that are not reflected in objective SES markers, making them a valuable tool for understanding and addressing mental health disparities.

## Supplementary material

10.1136/bmjopen-2025-111941 online supplemental file 1

## Data Availability

No data are available.
