# Supplementary material for: Associations of perceived family economy, registry-based parental education and income with adolescent psychological distress: the Young-HUNT cross-sectional studies 2006–2008 and 2017–2019
Source: BMJ Open. 2026 Jun 10;16(6):e111941. doi: 10.1136/bmjopen-2025-111941 (PMC13264949; doi:10.1136/bmjopen-2025-111941)
Supplement: online supplemental file 1 [file bmjopen-16-6-s001.docx]

# Supplementary:

| **Supplementary table 1: ^1^**Income range and median, the Young-HUNT3 Survey (2006–2008) and the Young-HUNT4 Survey (2017–2019), in the complete sample. | | |
| --- | --- | --- |
| **Income in the Young-HUNT3 Survey (nkr):** | | |
| **Income mother:** | **Income range:** | **Median:** |
| Low | 0 - 224 877 | 194 577 |
| Middle | 224 885 - 276 311 | 248 927 |
| High | 276 350 - 1 518 269 | 320 618 |
| **Income father:** |  |  |
| Low | 0 - 231 540 | 200 986 |
| Middle | 231 631 - 283 493 | 255 857 |
| High | 283 513 - 1 522 303 | 331 221 |
| **Income in the Young-HUNT4 Survey (nkr):** | | |
| **Income mother:** |  |  |
| Low | 0 - 344 919 | 295 692 |
| Middle | 344 926 - 432 819 | 385 643 |
| High | 432 994 - 6 157 360 | 497 759 |
| **Income father:** |  |  |
| Low | 0 - 356 608 | 313 412 |
| Middle | 356 649 - 447 350 | 397 432 |
| High | 447 379 - 8 712 774 | 513 650 |
| *Note.* ^1^Income = total household income, after tax, divided by the number of family members in the household. | | |

| **Supplementary table 2:** Definition parental education (from Statistics Norway). | | |
| --- | --- | --- |
| **Education: mother/father** | **Definition:** | **Years:** |
| IU: Unknown or no completed education | **Primary**  **Education** | **<10 years** |
| GS: Basic school level | **Primary**  **Education** |  |
| VGS: Upper secondary education | **Secondary**  **Education** | **10-14 years** |
| FS: Tertiary vocational education | **Secondary**  **Education** |  |
| UHK: Higher education, short | **Tertiary**  **Education** | **>14 years** |
| UHL: Higher education, long | **Tertiary**  **Education** |  |

| **Supplementary Table: 3:** Descriptive characteristics: the Young-HUNT3 Survey (2006–2008) and the Young-HUNT4 Survey (2017–2019): ^1^Young-HUNT sample and ^2^HADS-sample. | | | | |
| --- | --- | --- | --- | --- |
| **Survey:** | **Young-HUNT3:** | | **Young-HUNT4:** | |
| **Sample:** | **^1^Young-HUNT** | **^2^HADS** | **^1^Young HUNT** | **^2^HADS** |
| **Sample size (N):** | 7494 | 2077 | 7539 | 1704 |
| **Girls (N (%)):** | 3856 (51.5) | 1035 (49.8) | 3882 (51.5) | 827 (48.5) |
| Mean age (SD) | 15.9 (1.8) | 16.0 (1.8) | 16.1 (1.8) | 16.2 (1.8) |
| Mean HSCL-5 total score (SD) | 1.7 (0.6) | 1.6 (0.6) | 1.9 (0.8) | 1.9 (0.7) |
| Worse self-perceived family financial stress (N (%)): | 396 (10.3) | 70 (6.8) | 358 (9.2) | 59 (7.1) |
| **Boys (N (%)):** | 3638 (48.6) | 1042 (50.2) | 3657 (48.5) | 877 (51.5) |
| Mean age (SD) | 15.9 (1.7) | 16.0 (1.7) | 16.1 (1.8) | 16.2 (1.8) |
| Mean HSCL-5 total score (SD) | 1.3 (0.4) | 1.3 (0.4) | 1.4 (0.5) | 1.4 (0.5) |
| Worse self-perceived family financial stress (N (%)): | 304 (8.4) | 67 (6.4) | 241 (6.6) | 25 (2.9) |
| *Note. ^1^* Adolescents participating in the Young-HUNT3 Survey and the Young-HUNT4 Survey, ^2^ Sub sample of the complete sample: Adolescents with two parents participated in the HUNT3 or HUNT4 Survey with HADS scores. | | | | |

| **Supplementary table 4:** The Young-HUNT3 survey (2006–2008) and the Young-HUNT4 Survey (2017–2019). Relative difference (RD): SES and psychological distress among girls and boys (13–19 years) in a sub-sample from the ^1^complete sample and adjusting for HADS (red numbers). | | |
| --- | --- | --- |
| **Survey:** | **The Young-HUNT3 Survey**  (N=2077) | **The Young-HUNT4 Survey**  (N=1704) |
|  | **N (%)** | **N (%)** |
| **Girls:** | 1035 (49.83) | 827 (48.53) |
|  | **RD (95%CI)** | **RD (95%CI)** |
| **Self-perceived family economy:** |  |  |
| Worse (ref same) | 1.12 (1.04-1.21)  1.12 (1.03-1.20) | 1.23 (1.12-1.35)  1.22 (1.11-1.33) |
| Better (ref same) | 1.00 (0.95-1.06)  1.00 (0.95-1.06) | 1.01 (0.95-1.08)  1.01 (0.95-1.08) |
| ^2^**Income mother:** |  |  |
| Middle (ref low) | 1.06 (0.99-1.14)  1.07 (0.99-1.14) | 1.01 (0.93-1.09)  1.01 (0.94-1.10) |
| High (ref low) | 0.97 (0.89-1.05)  0.98 (0.90-1.06) | 0.99 (0.90-1.10)  1.00 (0.90-1.10) |
| ^2^**Income father:** |  |  |
| Middle (ref low) | 1.01 (0.94-1.08)  1.01 (0.94-1.08) | 1.01 (0.93-1.09)  1.01 (0.93-1.09) |
| High (ref low) | 1.11 (1.02-1.21)  1.12 (1.03-1.22) | 1.01 (0.92-1.12)  1.02 (0.92-1-12) |
| **Education mother:** |  |  |
| Secondary education  (ref primary) | 1.05 (0.97-1.12)  1.05 (0.98-1.12) | 1.06 (0.93-1.22)  1.07(0.93-1.23) |
| Tertiary education  (ref primary) | 1.06 (0.99-1.14)  1.08 (1.00-1.16) | 1.03 (0.90-1.18)  1.04(0.91-1.19) |
| **Education father:** |  |  |
| Secondary education  (ref primary) | 0.97 (0.91-1.03)  0.97 (0.92-1.04) | 1.11(1.00-1.25)  1.12(1.00-1.25) |
| Tertiary education  (ref primary) | 0.99 (0.93-1.07)  1.01 (0.94-1.08) | 1.08 (0.96-1.21)  1.08(0.96-1.22) |
|  | **N (%)** | **N (%)** |
| **Boys:** | 1042(50.17) | 877(51.47) |
| **Self-perceived family economy:** |  |  |
| Worse (ref same) | 1,23 (1.15-1.32)  1.22 (1.14-1.30) | 1.02 (0.90-1.15)  1.02 (0.90-1.14) |
| Better (ref same) | 1.03 (0.99-1.07)  1.03 (0.99-1.07) | 1.02 (0.98-1.07)  1.02 (0.98-1.07) |
|  | **RD (95%CI)** | **RD (95%CI)** |
| ^2^**Income mother:** |  |  |
| Middle (ref low) | 0.98 (0.93-1.04)  0.99 (0.93-1.05) | 1.10 (1.03-1.17)  1.11 (1.03-1.18) |
| High (ref low) | 0.93 (0.87-1.00)  0.94 (0.88-1.01) | 1.04 (0.97-1.12)  1.05 (0.97-1.13) |
| ^2^**Income father:** |  |  |
| Middle (ref low) | 1.00 (0.95-1.06)  1.00 (0.95-1.06) | 0.95 (0.89-1.01)  0.95 (0.89-1.01) |
| High (ref low) | 1.10 (1.02-1.18)  1.10 (1.02-1.18) | 0.97 (0.90-1.04)  0.97 (0.91-1.05) |
| **Education mother:** |  |  |
| Secondary education  (ref primary) | 0.97 (0.92-1,03)  0.98 (0.93-1.04) | 0.96 (0.86-1.08)  0.98 (0.87-1.10) |
| Tertiary education  (ref primary) | 0.97 (0.92-1.02)  0.98 (0.93-1.04) | 0.97 (0.86-1.08)  0.99 (0.89-1.11) |
| **Education father:** |  |  |
| Secondary education  (ref primary) | 1.05 (0.99-1.01)  1.05 (0.99-1.11) | 0.89 (0.81-0.98)  0.88 (0.80-0.97) |
| Tertiary education  (ref primary) | 1.03 (0.97-1.09)  1.03 (0.97-1.10) | 0.91 (0.82-1.00)  0.91 (0.82-1.01) |
| *Note.* ^1^Adolescents with two parents participated in the HUNT3 or HUNT4 Survey: mother and father in the same model,^2^Income  = total household income, after tax, divided by the number of family members in the household. | | |

| **Supplementary Table 5: Sensitivity analysis:** Relative difference (RD): SES and psychological distress among girls and boys (13–19 years) in the Young-HUNT3 Survey (2006–08) and the Young-HUNT4 Survey (2017–19), ^1^Complete sample (black numbers) and Complete sample after excluding participants with parents of non-Norwegian backgrounds (red numbers): | | |
| --- | --- | --- |
| **Survey:** | **The Young-HUNT3 Survey:** | **The Young-HUNT4 Survey:** |
|  | RD (95%CI): | RD (95%CI): |
| **Girls:** | 2576/2342 | 2328/2084 |
| **Self-perceived family financial stress:** |  |  |
| Worse (ref same) | 1.21 (1.15-1.27)  1.21(1.15-1.27) | 1.24 (1.17-1.31)  1.20(1.13-1.28) |
| Better (ref same) | 1.03 (0.99-1.06)  1.03(1.00-1.07) | 1.03 (0.99-1.07)  1.03(0.99-1.07) |
| ^2^**Income mother:** |  |  |
| Middle (ref low) | 1.04 (1.00-1.09)  1.02(0.98-1.07) | 0.97 (0.92-1.01)  0.99(0.94-1.03) |
| High (ref low) | 0.95 (0.91-1.00)  0.96(0.91-1.00) | 0.94 (0.89-0.99)  0.97(0.91-1.02) |
| ^2^**Income father:** |  |  |
| Middle (ref low) | 1.01 (0.97-1.05)  1.03(0.98-1.07) | 1.00 (0.95-1.04)  1.00(0.96-1.05) |
| High (ref low) | 1.10 (1.05-1.15)  1.11(1.06-1.16) | 1.00 (0.95-1.05)  1.00(0.94-1.05) |
| **Education mother:** |  |  |
| Secondary education (ref primary) | 1.00 (0.97-1.05)  1.01(0.97-1.05) | 0.97 (0.90-1-03)  0.98(0.91-1.06) |
| Tertiary education (ref primary) | 1.02 (0.97-1.06)  1.02(0.97-1.06) | 0.93 (0.87-0.99)  0.95(0.88-1.02) |
| **Education father:** |  |  |
| Secondary education (ref primary) | 0.99 (0.95-1.03)  0.98(0.94-1.02) | 1.00 (0.94-1.05)  1.01(0.95-1.08) |
| Tertiary education (ref primary) | 0.98 (0.94-1.03)  0.97(0.93-1.02) | 0.97 (0.91-1.03)  1.00(0.93-1.06) |
| **Boys:** | 2534/2314 | 2308/2029 |
| **Self-perceived family financial stress:** |  |  |
| Worse (ref same) | 1.23 (1.18-1.28)  1.23(1.17-1.29) | 1.16 (1.09-1.23)  1.14(1.07-1.22) |
| Better (ref same) | 1.03 (1.00-1.05)  1.03(1.00-1.06) | 1.02 (0.99-1.05)  1.02(0.99-1.06) |
| ^2^**Income mother:** |  |  |
| Middle (ref low) | 1.01 (0.97-1.04)  1.00(0.97-1.04) | 0.99 (0.95-1.03)  1.02(0.97-1.06) |
| High (ref low) | 0.98 (0.94-1.02)  0.98(0.94-1.02) | 0.99 (0.94-1.04)  0.98(0.94-1.03) |
| ^2^**Income father:** |  |  |
| Middle (ref low) | 0.98 (0.95-1.02)  0.98(0.95-1.02) | 0.99 (0.95-1.03)  0.98(0.94-1.02) |
| High (ref low) | 1.03 (0.99-1.07)  1.03(0.99-1.07) | 1.01 (0.96-1.06)  1.02(0.97-1.07) |
| **Education mother:** |  |  |
| Secondary education (ref primary) | 0.98 (0.95-1.02)  0.99(0.95-1.02) | 0.98 (0.92-1.04)  0.98(0.92-1.04) |
| Tertiary education (ref primary) | 0.99 (0.96-1.03)  0.99(0.96-1.03) | 0.98 (0.93-1.04)  0.98(0.92-1.04) |
| **Education father:** |  |  |
| Secondary education (ref primary) | 1.01 (0.98-1.04)  1.02(0.99-1.06) | 0.99 (0.94-1.05)  1.00(0.95-1.06) |
| Tertiary education (ref primary) | 1.00 (0.96-1.04)  1.01(0.97-1.05) | 1.00 (0.94-1.06)  1.01(0.95-1.08) |
| *Note. ^1^*Adolescents with two parents participated in the HUNT3 or HUNT4 Survey; mother and father in the same model,^2^Income = total household income, after tax, divided by the number of family members in the household; RD = Relative difference. 95%CI = 95 percent Confidence interval. | | |
