# Supplementary material for: Associations of perceived family economy, registry-based parental education and income with adolescent psychological distress: the Young-HUNT cross-sectional studies 2006–2008 and 2017–2019
Source: BMJ Open. 2026 Jun 10;16(6):e111941. doi: 10.1136/bmjopen-2025-111941 (PMC13264949; doi:10.1136/bmjopen-2025-111941)
Supplement: Supplementary file 2 [file bmjopen-2025-111941.reviewer_comments.pdf]

PEER REVIEW HISTORY

BMJ Open publishes all reviews undertaken for accepted manuscripts. Reviewers are asked to complete a checklist review form and are provided with free text boxes to elaborate on their assessment. These free text comments are reproduced below.

ARTICLE DETAILS

Title (Provisional)

The associations of perceived family economy, registry based parental education and income with adolescent psychological distress. The Young-HUNT cross-sectional studies 2006-08 and 2017-19.

Authors

Selvik , Karoline Louise Imingen Imingen; Strandås, Bent Martin Eliassen; Olsson, Craig A; Braaten, Tonje; Bjerkeset, Ottar

---

VERSION 1 - REVIEW

**Reviewer**	**1**
**Name**	**Dack, Kyle**
**Affiliation**	**University of Bristol, MRC Integrative Epidemiology Unit**
**Date**	**30-Nov-2025**
**COI**	**None**

Statistical review:

The methodological approach and its interpretation are reliable, and overall the paper is clear and adheres well to STROBE guidelines.

My only concern is that the analysis involves pooling data from two significantly different time periods – 2006-2008 and 2017-2019. There is not much discussion of the comparability of these two datasets, or exploration of whether they are suitable to analyse in this way. Additionally I am unsure if parental income was adjusted for inflation – otherwise this will add significant measurement error or skew to the tertiles which could have obscured the detection of a true association. I suggest these issues are explored more in the analysis and/or discussion.

Abstract:

1. No comments or suggestions regarding the abstract – it is clear and contains all essential information.

Introduction:

2. The introduction states that mental disorder prevalence in Western European children aged 10-19 increased from 20% to 23%. I wanted to find the source of this, but reference 6 (VizHuv) is challenging to use. It requires users to sign-up, and within the tool I could not find (a) an age band of 10-19 (only 5-14 and 15-49 are offered) nor (b) prevalence statistics (only YLD or DALYs are available). I suggest either citing a more specific reference, or add more information so that readers can locate the referenced data.

Methods:

3. Dependent variable: “The average HSCL-5 score” I suggest rephrasing to “mean HSCL-5 score” to avoid ambiguity regarding mean/median.

4. Independent variables and covariates: It would be helpful if possible to distinguish the data source for these - which came from Young-Hunt and which from adult-HUNT. It is possible for readers to guess, e.g. I assume parental income is from adult HUNT, but it would be clearer if the authors could confirm this in-text.

5. Parental income: There is approximately 10 years between HUNT-3 and HUNT-4, and inflation would impact the comparison of the reported income and tertile membership (HUNT-4 members are likely to be a larger proportion of the upper tertile). I’m unsure if adjustment was made for inflation – I suggest adding it to the methods, or if not performed, consider correcting for it.

6. Statistical analysis: I suggest adding more information regarding the modelling strategy and variables included.

(a) The use of mixed-effect modelling – what was the data structure being accounted for, and what parameters (e.g. random slope) were used to incorporate it? I think that it may be to account for multiple family members being within the sample, but if so it would be clearer to state this and define it (e.g. if cousins count).

(b) Could the full model specification be listed. Currently 3 independent variables are reported, but not the covariates parental country of origin and parental anxiety/depression.

(c) Secular trend analysis – which times were compared how specifically was this evaluated?

7. Given that the data contains two time periods with quite a large gap between them (2006-2008, 2017-2019), and there is evidence of variance between HUNT-3 and HUNT-4 both in the exposures and outcomes (tables 1, 2), did the authors consider evaluating the impact of this on model results, e.g. by stratifying the models or adding time as a covariate?

Results

8. Descriptive results – it would be helpful to remind readers of the maximum possible HSCL-5 score when reporting the mean values for boys and girls.

9. “Adjusting for parental mental health, did not alter the estimates. Additionally, there was little evidence for a secular change in the association between income and HSCL-5 total scores from the Young-HUNT3 to the Young-HUNT4 Survey.” I recommend adding specific statistics to support these and similar claims, e.g. the point estimate and 95% CI, or LR test result.

10. Sensitivity analysis: As above. Adding the full results as a supplementary table would help to support the reported lack of difference.

Discussion

11. The interpretation and discussion of limitations is reasonable and supported by the data – no suggestions.

**Reviewer**	**2**
**Name**	**Yasin, Siti Munira**
**Affiliation**	**University Technology MARA, Population Health and Preventive Medicine**
**Date**	**30-Dec-2025**
**COI**	**None**

Comment to author:

This manuscript addresses an important and timely public health issue: the rise in adolescent psychological distress and its relationship with socioeconomic status (SES), using high-quality population-based data from the Young-HUNT and HUNT studies linked with national registry data.

Introduction:

- The family stress model and family investment model were introduced here, but their relevance to subjective versus objective SES is not fully developed. Suggest to link this two aspects.

- How will this results affect the policy of Norway? What are the current strategies implemented to improve this issue and are they adequate? This can be then later discussed in the discussion sections.

Methodology

- Please clarify- clarity of survey- how many schools were involved in the survey, were they voluntary survey and were they school based? And how were they sampled/ recruited?

- Were they online survey and using any particular survey methods?

- Were the tools validated tools e.g. self-perceived financial stress? Please include its validation, and are they in English?

Results and discussion:

- There were too many repetitions of results from the table 1. Suggest to summarize these in the results section.

- One of the findings were lack of association between parental education and psychological distress- it is explained- but remains descriptive. Please describe more critically for the null findings, example include the income inequality, limitations in household income measurements, are there and effects of educational attainment over time, is there are particular buffering effects of the welfare state?

- Although author concluded that there were no secular trends of SES and psychological distress, but does it affect sensitivity of the test or power of study? Please discuss on this in discussion.

- Authors may consider adjusting for parental anxiety and depression, in a subsample of the complete data. The authors should more clearly in discussing the restriction and its possible biases. Suggest to perhaps include the comparison of characteristics between the full sample and the HADS-adjusted subsample which would add transparency.

- The various tools used in this area without clinical confirmation may be a setback, what can be done to improve this in the future and relating it to actionable strategies?

- Minor grammatical and stylistic edits is suggested. This would further improve readability (e.g. there were some missing spacing around percentages).

VERSION 1 - AUTHOR RESPONSE

Reviewer: 1

Dr. Kyle Dack, University of Bristol

Comments to the Author:

Statistical review:

The methodological approach and its interpretation are reliable, and overall the paper is clear and adheres well to STROBE guidelines.

My only concern is that the analysis involves pooling data from two significantly different time periods – 2006-2008 and 2017-2019. There is not much discussion of the comparability of these two datasets, or exploration of whether they are suitable to analyse in this way.

Response: Thank you for the valuable input. We have revised the manuscript title and aim to clarify that we compare data from two separate Young HUNT surveys (separate cohorts) rather than pooling them, in order to identify any evidence of a secular change in the risk relationship between self-reported family financial stress and registry based parental SES markers (household income and parental education) and adolescent anxiety and depression symptoms, within a descriptive model.

Revision, Introduction, aim: "*In this study, we aim to investigate the associations of adolescents' self-reported family financial stress and registry based parental income and education, with adolescent anxiety and depression symptom level. Secondly, we examined potential secular changes in these associations. Data were drawn from the Young-HUNT study and the HUNT Study in 2006-2008 and 2017-2019, and Statistics Norway*".

Additionally I am unsure if parental income was adjusted for inflation – otherwise this will add significant measurement error or skew to the tertiles which could have obscured the detection of a true association. I suggest these issues are explored more in the analysis and/or discussion.

Response: We divided parental income into tertiles (separately for 2006-2008 and 2017-2019*),* to address the challenge of inflation. Instead of comparing absolute values (which may be influenced by inflation). Refer also to response in point 5.

Abstract:

1. No comments or suggestions regarding the abstract – it is clear and contains all essential information.

Response: Thank you. Of note, we have now rewritten and structured the Abstract in accordance with the BMJ Open Guidelines.

Introduction:

2. The introduction states that mental disorder prevalence in Western European

children aged 10-19 increased from 20% to 23%. I wanted to find the source of this, but reference 6 (VizHuv) is challenging to use. It requires users to sign-up, and within the tool I could not find (a) an age band of 10-19 (only 5-14 and 15-49 are offered) nor (b) prevalence statistics (only YLD or DALYs are available). I suggest either citing a more specific reference, or add more information so that readers can locate the referenced data.

Response: Thank you, we understand that this information was not straightforward for the reader to access. In the revised version, we edited the text and added new references.

Revision: "*Simultaneously, the prevalence and impact of mental disorders in 10-19 years olds increased markedly from 1990 to 2021* [1,6,7]."

References:

1. McGorry PD, Mei C, Dalal N, Alvarez-Jimenez M, Blakemore S-J, Browne V, et al. The Lancet Psychiatry Commission on youth mental health. The Lancet Psychiatry. 2024;11(9):731–74.

6. Tian J, Yan N, Hu X, Tian S, Wang Y, Mackay LE, et al. Global burden of mental disorders among adolescents and young adults, 1990–2021: a systematic analysis of the Global Burden of Diseases Study 2021. General Psychiatry. 2025;38(6):e102278.

7. Sappenfield O, Alberto C, Minnaert J, Donney J, Lebrun-Harris L, Ghandour R. National Survey of Children's Health Data Briefs [Internet]. Adolescent Mental and Behavioral Health, 2023. 2024 [10.02.26]. Available from: https://www.ncbi.nlm.nih.gov/books/NBK608531/#top.

Methods:

3. Dependent variable: "The average HSCL-5 score" I suggest rephrasing to "mean HSCL-5 score" to avoid ambiguity regarding mean/median.

Response: Thank you for the clarification. In the revised version, we edited the text as suggested.

Revision: "*The mean HSCL-5 score was calculated by dividing the total sum by five, with scores ranging from 1 to 4*".

4. Independent variables and covariates: It would be helpful if possible to distinguish the data source for these - which came from Young-Hunt and which from adult-HUNT. It is possible for readers to guess, e.g. I assume parental income is from adult HUNT, but it would be clearer if the authors could confirm this in-text.

Response: We agree that data sources should be clearly stated in the text. We have already included the data source in the description for each variable, e.g:

Self-perceived family financial stress: "*Self-perceived family financial stress was measured in both Young-HUNT Surveys using the question …*".

Self-reported psychological distress: "*Self-reported psychological distress (anxiety and depression symptoms) was assessed by a validated, reliable and widely used Norwegian short version of the Hopkins Symptom Checklist (HSCL), the five-item HSCL-5 in Young-HUNT3 and Young-HUNT4…*".

Parental income: "*This data was obtained from Statistics Norway for the same year the youth participated in Young-HUNT*".

Parental education level: "*Parental educational level was obtained from Statistics Norway for the same year the adolescent participated in Young-HUNT3 and Young-HUNT4.*".

Parental anxiety and depression symptoms: "*Parental anxiety and depression symptoms were assessed using the validated, reliable and widely used 14-item Hospital Anxiety and Depression rating Scale (HADS) in both HUNT Surveys…*".

Parental country of origin: "*Parental country of origin was obtained from Statistics Norway and categorized as…*".

5. Parental income: There is approximately 10 years between HUNT-3 and HUNT-4, and inflation would impact the comparison of the reported income and tertile membership

(HUNT-4 members are likely to be a larger proportion of the upper tertile). I'm unsure if adjustment was made for inflation – I suggest adding it to the methods, or if not performed, consider correcting for it.

Response: I am sorry if we have been unclear regarding the design. Instead of comparing absolute values (which may be influenced by inflation), we divided total household income into low, middle and high income using tertiles separate for The Young-HUNT3 Survey (2006–2008) and The Young-HUNT4 Survey (2017–2019) (supplementary Table 1). To our best knowledge, this should address the challenge of inflation adequately. Parental income was therefore not adjusted for inflation. We have implemented minor revisions to the wording in paragraph 2 page 5 to enhance clarity.

Revision: "*We divided total household income into low, middle and high income using tertiles separate for Young-HUNT3 (2006–2008) and Young-HUNT4 (2017–2019) (supplementary Table 1)*".

6. Statistical analysis: <u>I suggest adding more information regarding the modelling strategy and variables included.</u>

(a) The use of mixed-effect modelling – what was the data structure being accounted for, and what parameters (e.g. random slope) were used to incorporate it? I think that it may be to account for multiple family members being within the sample, but if so it would be clearer to state this and define it (e.g. if cousins count).

Response: Thank you for the clarification. In the revised version, we edited the text.

Revision: "*Associations between self-perceived family financial stress and parental income and education, and adolescent anxiety and depression symptom levels were examined using a multilevel mixed-effects generalized linear model with random intercept to account for correlation between siblings*".

(b) Could the full model specification be listed. Currently 3 independent variables are reported, but not the covariates parental country of origin and parental anxiety/depression.

Response: We agree on reporting estimates adjusted for parental anxiety and depression and have included them in a supplementary table (Supplementary table 4).

Further, we notice that our heading "Covariates" on page 5 is misleading. Parental country of origin is not a covariate. This variable was only used in a sensitivity analysis, where we repeated the main analysis on the complete sample after excluding participants with parents of non-Norwegian backgrounds. We now report these results in a separate new supplementary table (Supplementary table 5).

Revision: We changed our heading on page 5, paragraph 2 to "Additional variables" and created a new supplementary table (Supplementary table 5).
Next, we revised the text to improve clarity and coherence:

"*A gamma distribution with a log link function was used to estimate the ratio of predicted means as a measure of relative differences (RD) with 95% confidence intervals (95% CIs), stratified by gender. Analyses were adjusted for continuous age. Since the number of missing HADS-T scores were relatively high, despite extrapolation, we adjust for parental HADS-T only in a subsample of the "Complete sample"; the "HADS-sample".*

Parental country of origin was used in a sensitivity analysis to examine potential changes in the association between the three SES factors and symptom level (mean HSCL-5 total score) after excluding participants with parents of non-Norwegian background from the "Complete sample"".

(c) Secular trend analysis – which times were compared how specifically was this evaluated?

Response: We compare secular change (not secular trends) using data from two cross-sectional surveys: The Young-HUNT/HUNT3 Survey (2006–2008) and The Young-HUNT/HUNT4 Survey (2017–2019).

7. Given that the data contains two time periods with quite a large gap between them (2006-2008, 2017-2019), and there is evidence of variance between HUNT-3 and HUNT-

4 both in the exposures and outcomes (tables 1, 2), did the authors consider evaluating the impact of this on model results, e.g. by stratifying the models or adding time as a covariate?

Response: Based on the reviewers comments, we recognize that the text may lack clarity regarding the analysis. We have therefore included additional information to provide a more precise and detailed description of our approach.

Revision: Refer to the response to comment 6B and the text revision in paragraph 3 under Statistical Analyses: "*Secular change from Young-HUNT3 to Young-HUNT4 was examined using the likelihood ratio test to compare models with and without terms for interaction between survey and SES factors*".

Results:

8. Descriptive results – it would be helpful to remind readers of the maximum possible HSCL-5 score when reporting the mean values for boys and girls.

Response: Thank you for the clarification. In the revised version, we edited the text accordingly.

Revision: "*The mean HSCL-5 total score, with the maximum possible score of 4, was 1.6 for girls in Young-HUNT3 and 1.9 points in Young-HUNT4. The respective numbers for boys were 1.3 and 1.4 points*".

9. "Adjusting for parental mental health, did not alter the estimates. Additionally, there was little evidence for a secular change in the association between income and HSCL-5 total scores from the Young-HUNT3 to the Young-HUNT4 Survey." I recommend adding specific statistics to support these and similar claims, e.g. the point estimate and 95% CI, or LR test result.

Response: During the revision process, we identified that references to Supplementary Table that demonstrates point estimates and 95%CI when adjusting for parental mental health were missing in the manuscript. This table Supplementary Table 4 has now been incorporated into the manuscript where appropriate.

Additionally, in the revised manuscript, we have included the statistically significant results from the likelihood ratio (LR) test.

10. Sensitivity analysis: As above. Adding the full results as a supplementary table would help to support the reported lack of difference.

Response: Thank you for your suggestion. We understand that a supplementary table would be a valuable addition and have included this in the revised version (refer to the file labelled "Supplementary copy" and our response to point 6b above).

Revision: "*Repeating the main analysis in the "Complete sample" after excluding participants with parents of non-Norwegian backgrounds did not alter the estimates (supplementary Table 5)"*.

Discussion:

11. The interpretation and discussion of limitations is reasonable and supported by the data – no suggestions.

Response: Thank you.

Reviewer: 2

Dr. Siti Munira Yasin, University Technology MARA, University of Malaya

Comments to the Author:

This manuscript addresses an important and timely public health issue: the rise in adolescent psychological distress and its relationship with socioeconomic status (SES), using high-quality population-based data from the Young-HUNT and HUNT studies linked with national registry data.

Introduction:

1. The family stress model and family investment model were introduced here, but their

relevance to subjective versus objective SES is not fully developed. Suggest to link this two aspects.

Response:

Thank you for this reminder. Since these two theories focus on different aspects of a family's financial situation, namely perceived financial stress (The family stress model, Cogan) and (Family investment model, Becker), it is of relevance to measures of SES in this study. We have revised this part of the Introduction accordingly.

Of note, we have now also touched upon this briefly in the Discussion part, regarding the different findings for subjective and objective measures of family SES.

Revision: «*The family stress model describes how perceived economic difficulties can create parental stress and behavioural changes that negatively impact family life, increasing the risk of worry and anxiety in children and adolescents [18]. In contrast, the family investment model centers upon how lack of objective, family material resources can limit children's opportunities to maintain health and achieve cognitive and educational development [19].*"

References [18-19]: Refer to manuscript.

2. How will this results affect the policy of Norway? What are the current strategies implemented to improve this issue and are they adequate? This can be then later discussed in the discussion sections.

Response: The study adds important knowledge to the body of literature on socioeconomic disparities in adolescent mental health in Norway. The study shows that subjective SES measures may be valuable tools for addressing mental health disparities in adolescents in Norway. However, it is beyond the scope of this paper to discuss current public health strategies and if our study results may affect governmental policies. Also, results from the two cross sectional surveys included in this study do not allow for conclusions regarding causal pathways linking socioeconomic factors and adolescent mental health. This study remains purely

descriptive, but can generate hypothesis for later studies using proper designs to elucidate these pathways.

Methodology

3. Please clarify- clarity of survey- how many schools were involved in the survey, were they voluntary survey and were they school based? And how were they sampled/ recruited?

Response: Thank you for pointing out the missing information. We have revised the text and added a new reference.

Revision regarding Young-HUNT, page 4:

"Adolescents recruited to this study consisted of two unique samples who participated in The Young-HUNT3 Survey (2006–2008, n=8199, 78.4% response rate) and The Young-HUNT4 Survey (2017–2019, n=8066, 76.0 % response rate). These studies are school-based surveys, and invitations were based on schools' class lists of all registered pupils primarily aged 13–19 years in all lower and upper Secondary Schools in the County. In the Young-HUNT3 survey (hereinafter referred to as Young-HUNT3) 66 schools participated and in the Young HUNT4 survey (hereinafter referred to as Young-HUNT4) 67 schools participated. Participants aged 16 and older provided informed written consent, while parents provided written consent for participants younger than 16 years. Adolescents completed the self-report questionnaire on paper in Young-HUNT3 and online in Young-HUNT4, during school hours. Pupils who were absent the day of the survey received the survey by post. Furthermore, clinical examinations were conducted on more than half of the participants [23, 24]".

References:

23. Rangul V, Holmen TL, Langhammer A, Ingul JM, Pape K, Fenstad JS, et al. Cohort Profile Update: The Young-HUNT Study, Norway. International Journal of Epidemiology. 2024;53(1).

24. Holmen TL, Bratberg G, Krokstad S, Langhammer A, Hveem K, Midthjell K, et al. Cohort profile of the Young-HUNT Study, Norway: A population-based study of adolescents. International Journal of Epidemiology. 2014;43(2):536–44.

Revision regarding HUNT, page 4:

"All inhabitants in Nord-Trøndelag aged 20 years and older were invited to participate in each survey of the HUNT Study. Participants completed a self-report questionnaire on paper or web based. Subsamples were invited to a short interview, new questionaries, clinical examinations and biological sampling [22]".

22. Åsvold BO, Langhammer A, Rehn TA, Kjelvik G, Grøntvedt TV, Sørgjerd EP, et al. Cohort Profile Update: The HUNT Study, Norway. International Journal of Epidemiology. 2023;52(1):e80–e91.

4. Were they online survey and using any particular survey methods?

Response: Please see our response above.

5. Were the tools validated tools e.g. self-perceived financial stress? Please include its validation, and are they in English?

Response: Questionnaires and instruments were distributed in Norwegian in the Young-HUNT and HUNT studies.

Self-perceived financial stress is a single item that originates from the WHO collaborative cross-national HBSC study in Europe. We used the Norwegian translation of this question and response alternatives from the official HUNT databank [1]. Of note, it has been translated and back translated according to standard procedures and is identical with the version used in previous HUNT studies (this information was integrated into the manuscript, and the corresponding reference was included).

The Norwegian version of the five-item HSCL-5 has been widely used in population studies, and was originally designed by Tambs et al [2] after thorough factor analyses. We included the reference confirming its validation.

The Norwegian version of the Hospital Anxiety and Depression Scale (HADS) has been widely used across patient- and study settings. It was validated in the HUNT 2 population and shows good psychometric properties [3]. We included the reference confirming its validation.

References [1-3]: Refer to the bottom of the document.

Revision: Self-perceived financial stress:

"*The question originates from the WHO collaborative cross-national Health Behaviour in School-aged Children (HBSC) study in Europe [28].*"

Reference:

28. HUNT-Databank. Socioeconomics Factors. 2024 [Available from: https://hunt-db.medisin.ntnu.no/hunt-db/variablegroup/Socioeconomics.

Revision: The five-item HSCL-5: the short version of the Hopkins Symptom Checklist (HSCL):

"*Self-reported psychological distress (anxiety and depression symptoms) was assessed by a validated, reliable and widely used Norwegian short version of the Hopkins Symptom Checklist (HSCL), the five-item HSCL-5 inYoung-HUNT3 and Young-HUNT4 [25-27].*"

References:

25. Strand BH, Dalgard OS, Tambs K, Rognerud M. Measuring the mental health status of the Norwegian population: A comparison of the instruments SCL-25, SCL-10, SCL-5 and MHI-5 (SF-36). Nordic Journal of Psychiatry. 2003;57(2):113–8.

26. Derogatis LR, Lipman RS, Rickels K, Uhlenhuth EH, Covi L. The Hopkins Symptom Checklist (HSCL): A self-report symptom inventory. Behavioral Science. 1974;19(1):1–15.
27. Tambs K, Moum T. How well can a few questionnaire items indicate anxiety and depression? Acta Psychiatrica Scandinavica. 1993;87(5):364–7.

Revison: The Hospital Anxiety and Depression Scale (HADS): We included the reference confirming its validation: "*Parental anxiety and depression symptoms were assessed using the validated, reliable and widely used 14-item Hospital Anxiety and Depression rating Scale (HADS) in both HUNT Surveys [29, 30].*"

30. Mykletun A, Stordal E, Dahl AA. Hospital Anxiety and Depression (HAD) scale: factor structure, item analyses and internal consistency in a large population. BrJPsychiatry. 2001;179:540–4.

Results and discussion:

6. There were too many repetitions of results from the table 1. Suggest to summarize these in the results section.

Response: We have attempted to limit repetitions and have now reduced the text in the section concerning descriptive analysis.

7. One of the findings were lack of association between parental education and psychological distress- it is explained- but remains descriptive. Please describe more critically for the null findings, example include the income inequality, limitations in household income measurements, are there and effects of educational attainment over time, is there are particular buffering effects of the welfare state?

Response:

We thank the reviewer for these interesting comments and questions. But as this study is purely descriptive, we cannot speculate on causal relationships. A different, analytical design would have been necessary to answer these questions. In the paper

we argue that low levels of income inequality and the social welfare system in Norway may partly explain the lack of statistical evidence between objective SES measures and mean HSCL-5 score in our study. We also write that subjective SES may reflect factors such as family wealth, neighbourhood socioeconomic conditions, past economic experiences, family composition, and the employment status of parents. However, as stated above, our study is not designed to address such research questions.

8. Although author concluded that there were no secular trends of SES and psychological distress, but does it affect sensitivity of the test or power of study? Please discuss on this in discussion.

Response: Of note, since we have only two measures about 10 years apart, we investigated possible secular change - not trends. Also, we claim our study should be exceedingly well powered to detect effects if they exist.

9. Authors may consider adjusting for parental anxiety and depression, in a subsample of the complete data. The authors should more clearly in discussing the restriction and its possible biases. Suggest to perhaps include the comparison of characteristics between the full sample and the HADS-adjusted subsample which would add transparency.

Response: Thank you for your valuable input. In the revised version we included a supplementary table (supplementary table 3) that compare the characteristics between the Young-HUNT sample and the HADS-adjusted subsample.

Revision: Descriptive results: "*The characteristics of the "HADS-sample" mirrored those of the other samples, with the notable exception that a smaller proportion of adolescents reported worse self-perceived family financial stress in the "HADS-sample" (supplementary Table 3)*".

Revision: Interpretation/main discussion, fifth paragraph: *"In our study, we adjusted the associations between self-perceived family financial stress and mean HSCL-5 total*

scores for parental anxiety and depression symptoms in a small subsample (the "HADS sample"). In the "HADS-sample", fewer adolescents reported worse self-perceived family financial stress compared to the original samples, yet the results remained unchanged".

10. The various tools used in this area without clinical confirmation may be a setback, what can be done to improve this in the future and relating it to actionable strategies?
Response: Young-HUNT and HUNT are population-based cohort studies, with the aim of informing public health policy (more so than clinical guidelines). Nevertheless, the outcome measure in Young-HUNT is the HSCL - an instrument commonly used across populations and healthcare settings, including specialised mental health services. The application of a standardised, psychiatric interview to verify clinical diagnosis would be ideal, but extremely costly and time consuming, and therefore not feasible in large scale studies such as Young-HUNT and HUNT. However, diagnostic interviews of a sub sample from these studies should be performed in the future waves of Young-HUNT and HUNT.

11. Minor grammatical and stylistic edits is suggested. This would further improve readability (e.g. there were some missing spacing around percentages).
Response: Ok.

Additional changes made by the main author and co-authors:

Abstract:

We have revised the abstract of our manuscript to conform to the preferred format of BMJ Open.

Results:

Figure 1: Minor wording change in the intermediate sample box within the flowchart for participation and exclusion in the Young-HUNT4 Survey from "HUNT3" to "HUNT4".

Table: 1: Minor wording change in the first column from "*Worse self-perceived family economy* (N(%)):" to "*Worse self-perceived family financial stress (N (%)):*"

Discussion:

During the review, we discovered that the third paragraph in the interpretation/main discussion was missing a reference.

Revision: "*The relationship between subjective SES and mental health demonstrated the largest effect size* [32]*.*"

Reference:

32. Quon, E.C. & McGrath, Subjective socioeconomic status and adolescent health: A meta-analysis. J.J. Health Psychology. 2014. Issue 5 Pages 433-447.

References:
1. HUNT-Databank. Socioeconomics Factors. 2024 [Available from: https://hunt-db.medisin.ntnu.no/hunt-db/variablegroup/Socioeconomics.
2. Tambs K, Moum T. How well can a few questionnaire items indicate anxiety and depression? Acta Psychiatrica Scandinavica. 1993;87(5):364–7.
3. Mykletun A, Stordal E, Dahl AA. Hospital Anxiety and Depression (HAD) scale: factor structure, item analyses and internal consistency in a large population. BrJPsychiatry. 2001;179:540–4.

VERSION 2 - REVIEW

**Reviewer**	**1**
**Name**	**Dack, Kyle**
**Affiliation**	**University of Bristol, MRC Integrative Epidemiology Unit**
**Date**	**26-May-2026**
**COI**	

The revision addressed all my queries, and I have no further suggestions.
